# Histone Deacetylase (HDAC) Inhibitors for the Treatment of Schistosomiasis

**DOI:** 10.3390/ph15010080

**Published:** 2022-01-10

**Authors:** Ehab Ghazy, Mohamed Abdelsalam, Dina Robaa, Raymond J. Pierce, Wolfgang Sippl

**Affiliations:** 1 Department of Medicinal Chemistry, Institute of Pharmacy, Martin-Luther-University of Halle-Wittenberg, 06120 Halle (Saale), Germany; ehab.ghazy@alexu.edu.eg (E.G.); mohamed.abdelsalam@pharmazie.uni-halle.de (M.A.); dina.robaa@pharmazie.uni-halle.de (D.R.); 2 Department of Pharmaceutical Chemistry, Faculty of Pharmacy, Alexandria University, Alexandria 21521, Egypt; 3 Centre d’Infection et d’Immunité de Lille, U1019—UMR9017—CIIL, Institute Pasteur de Lille, CNRS, Inserm, CHU Lille, Univ. Lille, F-59000 Lille, France; raymond.pierce@sfr.fr

**Keywords:** schistosomiasis, epigenetic, smHDAC8, hydroxamic acids, HDAC inhibitors, sirtuins

## Abstract

Schistosomiasis is a major neglected parasitic disease that affects more than 240 million people worldwide and for which the control strategy consists of mass treatment with the only available drug, praziquantel. Schistosomes display morphologically distinct stages during their life cycle and the transformations between stages are controlled by epigenetic mechanisms. The targeting of epigenetic actors might therefore represent the parasites’ Achilles’ heel. Specifically, histone deacetylases have been recently characterized as drug targets for the treatment of schistosomiasis. This review focuses on the recent development of inhibitors for schistosome histone deacetylases. In particular, advances in the development of inhibitors of *Schistosoma mansoni* histone deacetylase 8 have indicated that targeting this enzyme is a promising approach for the treatment of this infection.

## 1. Introduction

### 1.1. HDACs: Functions, Classes, and Therapeutic Potential

Histone acetylation is one of the most studied post-translational modifications. The state of histone acetylation is controlled through the “writers”, histone acetyltransferases (HATs) and the “erasers”, histone deacetylases (HDACs). HDACs are responsible for the removal of acyl (mostly acetyl) groups from lysine residues. Their substrates include histones and numerous non-histone proteins, such as p53, cytoskeleton proteins, RNA processing enzymes, and proteins involved in cell signaling and apoptosis [1]. So far, 18 different human HDACs isoforms have been identified, differing in size, cellular distribution, substrate, acyl group removed, and mechanism of catalytic activity. HDACs are classified into two major categories; the classical Zn^2+^-dependent HDACs and sirtuins [1]. Classical histone deacetylases, for which the abbreviation HDACs will refer from now on, comprise 11 enzymes that have a zinc ion in the active site responsible for their catalytic activity. These metalloenzymes have a conserved deacetylase domain but differ in size, cellular localization, and substrates [2]. They are further classified in classes I (HDACs 1–3, 8), IIa (HDACs 4, 5, 7, 9), IIb (HDACs 6, 10), and IV (HDAC11) [3,4,5]. Non-classical HDACs (class III) are named sirtuins due to their homology to the yeast silent information regulator 2 (SIR2). Unlike HDACs, the seven sirtuin isoforms exert their deacetylase activity by utilizing nicotinamide adenine dinucleotide (NAD^+^) as a cofactor; the catalytic reaction involves the cleavage of the nicotinamide moiety and the transfer of the acyl group from the lysine residue to ADP-ribose [6,7]. HDACs and sirtuins are involved in the regulation of different physiological functions, and their uncontrolled activity is linked to many pathological conditions. As a result, their structure, substrates, biological roles, and relation to disease have been extensively studied, and many informative reviews on HDACs (such as [1,8,9,10,11,12,13]) and sirtuins (such as [6,7,14,15,16]) are available. Moreover, HDAC inhibitors (HDACi) were investigated as potential therapeutic agents for several diseases [17,18,19,20,21,22,23,24], with oncology being the most successful field as six HDACi have received regulatory approval for the treatment of different hematological malignancies [1,25].

### 1.2. Repurposing Anticancer HDACi as Antiparasitic Agents

Neglected parasitic diseases affect millions of people with high morbidity and mortality. Lack of vaccines and a limited number of available drugs have resulted in their extensive use, raising the concern of resistance and consequent treatment failure. Additionally, current therapies sometimes involve long regimes, are usually active against only specific life-cycle forms of the parasites, and occasionally can have severe side effects [26]. As a result, new antiparasitic agents with novel mechanisms of action are urgently needed. One attractive strategy in this regard is the “piggyback” approach aiming to repurpose some drugs, already approved for other human diseases, as potential antiparasitic agents, which could decrease the time and costs to develop novel therapies [27]. Anticancer agents are especially attractive for this approach as tumors and parasites are similar in some aspects such as high metabolic and reproductive activity and the ability to survive within the host immune system [28]. Parasites are characterized by a complex life cycle with several morphologically distinct forms indicating the epigenetic control of gene expression. Indeed, major human parasites depend on HDACs and other epigenetic modulators for their survival and growth; therefore, HDACs were suggested as potential novel targets for antiparasitic therapy [26,29]. While the current work focuses on HDAC inhibitors as potential treatments for schistosomiases, HDACi for other parasitic infections such as malaria [30,31,32,33,34,35], trypanosomiasis [27,36,37,38], toxoplasmosis [39,40], leishmaniasis [41,42,43], and cestode infections [44] have also been reported and reviewed [26,29].

### 1.3. Schistosomiasis—Key Facts

Schistosomiasis is a parasitic infection that affects around 240 million people worldwide and is prevalent in poor tropical and subtropical regions, mostly in Africa [45]. The infection is caused by blood flukes from the genus *Schistosoma*, mainly *S. mansoni*, *S. haematobium*, and *S. japonicum* [45]. The parasite life cycle involves a specific snail intermediate host where the infective cercariae develop and then infect the human host to become the schistosomula (larvae) which later migrate to the liver and mature into adult male and female worms. Upon pairing, the eggs are then released in the feces to repeat the life cycle [46]. Current treatment and control of schistosomiasis exclusively depends on the antiparasitic agent praziquantel. Although this agent is active against parasitic flatworms, some reports suggested lack of efficacy during mass drug administration programs raising the concern of the development of drug resistance [46]. Therefore, new antischistosomal therapies with novel targets and mechanisms of action are of high interest. Repurposing of HDAC inhibitors against schistosomiasis appears a promising strategy given their previously mentioned success in the oncology field and also the identification of essential histone deacetylases in *S. mansoni* [47].

### 1.4. SmHDAC8, a Potential Drug Target in Schistosomes

Several orthologs of the human HDACs have been identified and characterized in schistosomes, belonging to classes I (smHDAC1, 3, and 8), II (smHDAC4, 5, and 6), and III (smSirt1, 2, 5, 6, and 7) [48,49]. Members of class I are expressed in all life cycles of the parasite, with the smHDAC8 isoform having the most abundant transcripts [50,51]. Interestingly, the human counterpart (hHDAC8) usually shows a lower level of expression in human cells compared to HDAC1 and 3 [51]. Therefore, it was suggested that this isoform may have a specific function for the parasite and could be an attractive target for novel antischistosomal therapy [50,51]. This was further supported by smHDAC8 knockdown studies demonstrating a significantly reduced viability and fertility of the parasites [52]. Crystal structures of smHDAC8 show that this enzyme adopts the canonical α/β HDAC fold, with specific solvent exposed loops corresponding to insertions in the schistosome HDAC8 sequence. These extensions do not affect the binding site and hence have no direct effect on the catalytic mechanism or ligand binding. SmHDAC8 shares the highest structural similarity with human HDAC8, and both enzymes show a characteristic subpocket in the binding site, dubbed the HDAC8-specific side pocket. This pocket is formed by the catalytic tyrosine residue, the L6 and L1-loop, where the latter loop is significantly shorter than in the other human HDAC isoforms. The HDAC8-specific pocket can hence be exploited for the development of selective inhibitors. When comparing the active sites of sm- and hHDAC8, few differences can be observed, with only one amino acid substitution in the binding site (Met274 in hHDAC8 is replaced by His292 in smHDAC8). Interestingly, crystal structures of smHDAC8 with several inhibitors showed that Phe151, which is located in the lysine binding channel, can adopt two different conformations, namely a flipped-in and a flipped-out conformation. The flipped-out conformation is characterized by Phe151 side chain turned away from the catalytic pocket and Lys20 instead pointing into the active site. This conformation is not found in resolved crystal structures of hHDAC isoforms and is considered to be unlikely also in hHDAC8 [52,53]. It is noteworthy that HDAC8 from other schistosome species (e.g., *S. haematobium, S. japonicum*), share a very similar catalytic site architecture, meaning that inhibitors of smHDAC8 will be likely to affect these species in the same way [48]. In Table 1, an overview of the interactions observed for reported smHDAC8 inhibitors in the catalytic pocket of the enzyme is given. 

## 2. Antischistosomal Effect of HDAC Inhibitors

### 2.1. Pan HDAC Inhibitors

The availability of HDAC inhibitors either as experimental probes or approved drugs was utilized to study phenotypic and molecular effects of HDAC inhibition in schistosomes. The HDAC inhibitor Trichostatin A (TSA) was shown to block the transformation of the free-swimming miracidia into the sporocyst in a concentration dependent manner [54]. From a therapeutic point of view, it is, however, more interesting to target the parasitic stages that live in the human host. Therefore, some HDAC inhibitors were also tested on both larvae and adult worms [55]. Again, treatment with TSA caused an increase in general levels of protein acetylation in schistosomes and induced mortality and apoptosis in schistosomula maintained in culture. Interestingly, the pan HDACi suberanilohydroxamic acid (SAHA, vorinostat) was not effective in that assay, while TSA was effective only after 2 days of treatment and at higher doses than those routinely used for cancer cell lines [55]. Moreover, another study revealed that some cellular functions, such as DNA replication and control of reactive oxygen species, were affected upon treatment of schistosomula with TSA which might explain the antischistosomal effect of this HDACi [47]. The FDA approved HDAC inhibitors, vorinostat (SAHA), belinostat, panobinostat, and romidepsin, were the major focus of another study as these anticancer agents were tested against several human parasites including Schistosoma mansoni [56]. The four compounds were not active against the schistosomula while panobinostat showed modest inhibition of adult worm pairing and egg production. In contrast, the cyclic tetrapeptide romidepsin showed complete inhibition of pairing and egg production at 10 µM concentration [56]. 

### 2.2. Selective smHDAC8 Inhibitors

#### 2.2.1. Hydroxamic Acid Based Inhibitors

**Figure 1:**
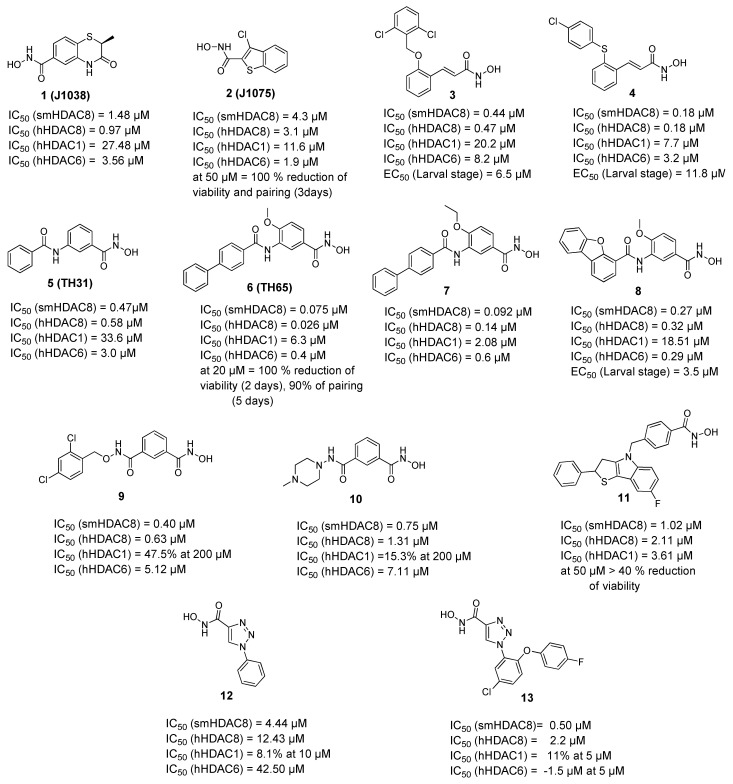
Examples of hydroxamic acid-based smHDAC8 inhibitors. IC_50_ values are cited for inhibition of the recombinant enzyme, EC_50_ values refer to viability testing on schistosomula.

A campaign was initiated based on a structure-based virtual screening approach where different commercially available derivatives bearing zinc binding groups were first docked to a homology model of smHDAC8 (later validated through solving the crystal structure) and the selected hits were tested in vitro for their inhibitory activity against schistosomal and human HDACs (smHDAC8, hHDAC1, hHDAC6, and hHDAC8 isoforms) [57]. Among the identified compounds, two hits showed good inhibitory activity against smHDAC8 in the low micromolar range (**1** and **2**, Figure 1). These two hits were then successfully cocrystallized with smHDAC8 and the crystal structure of the complex was solved [52,57]. It was also interesting that the 3-chlorobenzothiophene-2-hydroxamic acid **2** (J1075, Figure 1) exhibited dose-dependent killing of the schistosomula and adult worms as it induced 100% killing of schistosomula at 50 µM within 3 days and was also active at 10 µM. In addition, it was able to induce separation of the male and female worm pairs within 3 days at 50 µM and 5 days at 20 µM [52]. These two hits were then selected to serve as lead compounds for further structure-guided optimization to obtain more potent and selective smHDAC8 inhibitors.

To build on the previous results, a series of hydroxamic acid derivatives were designed and synthesized based on the general scaffold of the micromolar hit (**2**, J1075 [57]) [58]. To achieve better smHDAC8 activity and selectivity, different structural modifications were performed. For instance, the benzothiophene scaffold was changed into different bicyclic systems. Moreover, ring open analogues containing different substituted cinnamic-hydroxamic acid derivatives were synthesized. The newly synthesized compounds were evaluated for their inhibitory activity against schistosomal and major human HDACs isoforms. Several compounds showed potent activity against smHDAC8 ranging from the low micromolar to the nanomolar range. In addition, the most active compounds were further screened for lethality against the schistosomal larval stage. Interestingly, compounds **3** and **4** (Figure 1) showed significant and dose-dependent killing of the larvae with EC_50_ values of 6.5 and 11.8 µM, respectively, and markedly impaired egg laying of adult worm pairs maintained in culture [58].

In a further study, several benzhydroxamic acid derivatives were designed as open ring analogues of the previously reported hit (**1**, J1038 [57]) [59]. Structure-based design and chemical synthesis were combined to improve the activity against smHDAC8 and selectivity over major human HDAC isoforms. The developed inhibitors were tested in vitro for their inhibitory activity against schistosomal and human HDACs (smHDAC8, hHDAC1, hHDAC6, and hHDAC8 isoforms). Twenty-seven compounds demonstrated an inhibitory activity in the nanomolar range in the in vitro assays. Most of the designed compounds exhibited notable selectivity for smHDAC8 over the human HDAC isoforms tested (HDAC1 and HDAC6). Interestingly, some of these inhibitors also exhibited a preference for smHDAC8 over human HDAC8. In addition, phenotypic screening showed that compounds **6** and **7** caused a significant dose dependent killing of the schistosome larvae compared to praziquantel, which is known to be less active against larval developmental stages of the parasite [60]. Furthermore, at a concentration of 20 µM, compound **6** caused 90% separation of adult male and female worm pairs after 5 days. Moreover, compound **6** resulted in 80% reduction of egg laying of adult worm pairs at the same concentration [59]. It is worth mentioning that the cytotoxicity studies of the tested compounds against HEK293 (human embryonic kidney) cells showed that the compounds exhibit a relatively low effect on cell proliferation, which indicates that the inhibition of hHDAC8 does not induce intrinsic toxicity [59]. 

**Figure 2:**
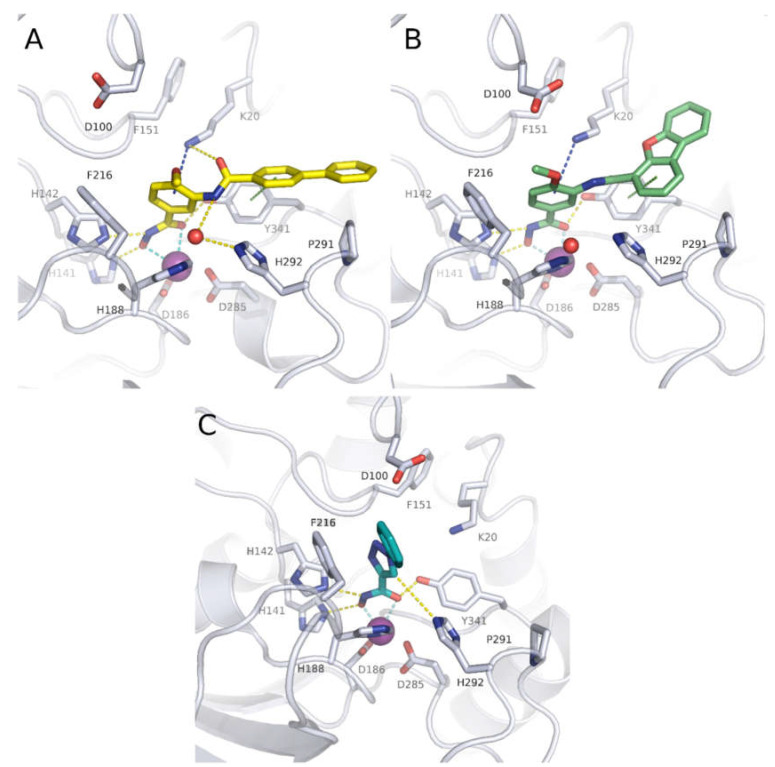
Crystal structures of some smHAC8 inhibitors. (**A**) Crystal structure of smHDAC8 with compound **6** shown as yellow sticks (PDB ID 5HTH); (**B**) crystal structure of smHDAC8 with compound **8** shown as green sticks (PDB ID 7P3S); (**C**) crystal structure of smHDAC8 with compound **12** shown as teal sticks (PDB ID 6TLD). The catalytic zinc ion is shown as purple sphere and water molecules as red spheres. Cyan-dashed lines indicate metal coordination, yellow-dashed line hydrogen bond interactions, green-dashed lines π-π interactions, and blue-dashed line cation-π interactions.

The crystal structure of compound **6** in complex with smHDAC8 showed a similar binding mode as that classically adopted by meta-substituted benzhydroxamic acid derivatives (Figure 2A) [53]. The hydroxamic acid moiety chelates the catalytic zinc ion in a bidendate fashion while undergoing a triad of hydrogen bonds with the catalytic tyrosine and two histidine residues. Phe151 shows a flipped-out conformation and the flipped in Lys20 displays a cation-π interaction with the central phenyl moiety. Meanwhile, the biphenyl capping group is positioned in the HDAC8-specific pocket, displaying π-π interactions with Tyr341 as well as hydrophobic interactions with Pro291.

In a following study, compound **6** was used as a scaffold to get more cellular active compounds against the parasite. The optimization process was guided by docking studies, and the new derivatives were designed through modifying the capping group by replacing the biphenyl ring system with polycyclic rings to target the hydrophobic HDAC8 specific pocket [61]. Several compounds showed potent smHDAC8 and hHDAC8 activity in the nanomolar range with decreased activity against hHDAC1 and 6. The most promising inhibitor, **8,** caused significant dose-dependent killing of the schistosome larvae with EC_50_ value of 3.5 µM and is thus the most potent antischistosomal HDAC inhibitor against this life stage reported so far. In addition, it caused noticeable impairment of egg laying of adult worm pairs. Finally, the developed compounds showed an acceptable safety profile on human HEK239 cells [61]. Inhibitor **8** was also cocrystallized with smHDAC8 confirming the conserved binding mode of benzhydroxamic acid derived inhibitors (Figure 2B). In parallel, the developed inhibitors were analyzed by docking and molecular dynamics simulation in order to rationalize the determined in vitro data [62].

A further study [63] was initiated based on the general scaffold of the previously reported benzhydroxamic acids such as compound **6** [59]. Briefly, a series of isophthalic acid-based HDAC inhibitors were designed as potential selective smHDAC8 inhibitors where a 3-acylbenzohydroxamic acid moiety was connected to different capping groups using an alkoxyamide group as a connecting unit [63]. The alkoxyamide group was previously identified [30,64] as a novel connecting unit which can probably enable charge assisted hydrogen bonds due to the additional polarization of the N–H bond. The work was then extended to include a hydrazide group as another connecting unit. Compounds **9** and **10** showed submicromolar activity against smHDAC8 with IC_50_ values 0.4 and 0.75 µM, respectively, and good selectivity over hHDAC1. Moreover, both compounds showed almost 10-fold selectivity over hHDAC6 and modest preference for smHDAC8 over hHDAC8. Unfortunately, the compounds were found to be inactive against the parasite in the cellular assay.

Another series of smHDAC8 inhibitors was designed based on structure-based virtual screening using High Throughput Docking (HTD) and phenotypical characterization of the selected hits [65]. The identified compounds had a hydroxamic acid group coupled to different capping groups such as (spiro)indoline or a tricyclic thieno[3,2-b]indole core [65]. Since the main goal of the study was to obtain compounds with drug like properties and strong activity against the parasite, these hits were then tested against the *S. mansoni* larval stage using an ATP-based viability assay. Seven compounds were found to reduce schistosomula viability in a dose dependant manner with EC_50_ values ranging from 13 to 50 µM under assay conditions. Moreover, further investigations were performed on other parasite developmental stages. Some of the compounds from both classes caused impaired viability of juvenile and adult worms, with compound **11** decreasing the viability of the treated adult *S. mansoni* worm pairs by more than 40% at 50 µM. In addition, some compounds were shown to impact egg production in vitro and induce morphological alterations of the adult schistosome reproductive systems. Some of the compounds showed activity against smHDAC8 in the low micromolar range [65].

Recently, novel triazole-based hydroxamic acids were identified as smHDAC8 inhibitors with improved selectivity for the smHDAC8 over the human orthologues (HDAC8, HDAC1, and HDAC6) [66]. Crystallographic studies of smHDAC8 complexed with **12** showed that the triazole ring of **12** makes a weak hydrogen bond with the smHDAC8 specific residue His292, an aromatic interaction with its imidazole ring as well as a π-cation interaction with Lys20 of smHDAC8 (Figure 2C). Interestingly, these previous interactions were not observed between **12** and the corresponding amino acids of the human orthologue HDAC8 as, in the case of hHDAC8, the His292 is replaced by Met274. Furthermore, the preferred orientation of the triazole moiety in smHDAC8 allows a π-stacking interaction between the phenyl ring of **12** and Phe216, whereas in the case of hHDAC8 this interaction with the corresponding Phe208 is not observed. To enhance the affinity of the triazole-hydroxamic acid hit **12** toward smHDAC8 and improve its selectivity, some structural modifications were performed on the phenyl ring of **12** by introducing a chlorine atom and an aromatic substituent at positions 5 and 2, respectively, which resulted in compound **13**. Interestingly, compound **13** was found to exhibit a superior smHDAC8 inhibitory activity (IC_50_ = 0.50 µM) compared to the lead compound **12** (IC_50_ = 4.44 µM). Comparison of the crystal structures of smHDAC8/**12** and smHDAC8/**13** revealed that compound **13** is slightly tilted within the active pocket of smHDAC8 compared to compound **12**, in which the triazole ring of **13** is closer to the smHDAC8 specific residue His292. Another observation is that the fluoro-phenyl capping group of **13** is stacked onto the Tyr341 side chain, which is considered as an essential interaction for improving the affinity toward smHDAC8. However, the promising in vitro smHDAC8 activity of the triazole compounds was not translated into schistosomicidal activity as they only showed low activity against the schistosomula, probably due to poor transport of the compounds across the parasite tegument [66].

#### 2.2.2. Non-Hydroxamic Acid Based Inhibitors

**Figure 3:**
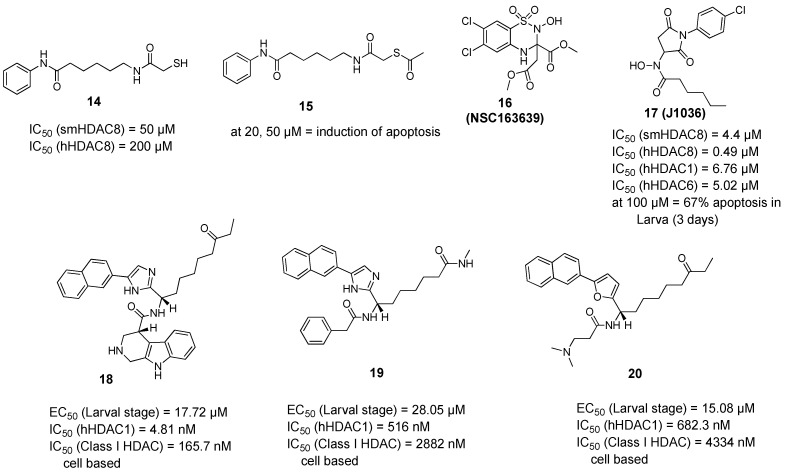
Examples of non-hydroxamic acid smHDAC8 inhibitors.

The quest for selective inhibitors of smHDAC8 started with a study in which a small-focused library of HDAC inhibitors was screened against the parasitic recombinant enzyme. Besides many active hydroxamic acids, an interesting mercaptoacetamide analogue of SAHA (**14**) was identified as a micromolar smHDAC8 inhibitor. Despite being less active on smHDAC8 than the other hydroxamic acid-based inhibitors, this thiol derivative showed a better selectivity towards hHDAC8 compared to SAHA [67]. To gain more insights, the cocrystallized structure of compound **14** bound to smHDAC8 was solved, representing the first example of a co-complex of histone deacetylases of any origin with a mercaptoacetamide. The crystal structure of smHDAC8 and **14** revealed that this thiol inhibitor is accommodated in the catalytic pocket, where it binds to both the catalytic zinc ion and the catalytic tyrosine Tyr341 via its mercaptoacetamide group. Interestingly, it was also shown that changing the zinc binding warhead results in a different positioning of the hydrophobic alkyl linker and the aromatic capping group common to compound (**14**) and SAHA, which might explain the differences in the activity and the selectivity of **14** toward smHDAC8 over hHDAC8 compared to SAHA. Taking into consideration the inapplicability of free thiol groups for cellular testing, an ester prodrug of the thiol was prepared (compound **15**, Figure 3) and tested against schistosomula. This prodrug induced dose- and time-dependent killing of cultured schistosomula in the range 10–50 µM, as well as induction of apoptosis at concentrations of 20 and 50 µM [67].

A virtual screening approach was reported [68] as the authors used the available smHDAC8-inhibitor complexes to develop a structure-based per-residue 3D QSAR (COMBINEr 2.0) model able to rationalize the crucial smHDAC8-ligand interactions. This model was used to screen the NCI Diversity Set V and identified a benzothiadiazine dioxide derivative (**16**, NSC163639). This compound showed a moderate in vitro smHDAC8 activity (37% inhibition at 30 µM) and some selectivity against human HDACs. To get a primary idea about the structure–activity relationship of this hit, two close analogues were synthesized and their smHDAC8 activity was tested and explained by means of docking studies. The authors suggested, as future work, that attachment of strong zinc binding groups such as a hydroxamic acid moiety through a linker instead of the ester groups in (**16**, NSC163639) could lead to a favorable placement and interactions of the benzothiadiazine moiety in the smHDAC8 active site [68].

Based on another docking-based virtual screening approach, another class of smHDAC8 inhibitors was identified [69]. Eight compounds having the general scaffold of N-(2,5-dioxopyrrolidin-3-yl)-n-alkylhydroxamic acid were identified and tested in vitro for their inhibitory activity against schistosomal and major human HDACs (smHDAC8, hHDAC1, hHDAC6, and hHDAC8 isoforms) [69]. The newly identified hits exerted a smHDAC8 inhibitory activity with IC_50_ values ranging from 4.4 to 20.3 µM. In addition, they showed activity against hHDAC1, 6, and 8. Among the identified compounds, (**17**, J1036) induced dose-dependent apoptosis in the *S. mansoni* larvae, affecting around 67% of the larvae after 3 days of incubation at a concentration of 100 µM, compared to the effect induced by SAHA (43% apoptosis at a dose of 100 µM). In addition, (**17**, J1036) was successfully cocrystallized with smHDAC8, which confirmed the in silico prediction of this compound. Analysis of the crystal structure of the smHDAC8/J1036 complex showed that the internal hydroxamic acid group was able to chelate the catalytic zinc ion in a bidentate manner, showing an inverted binding mode compared to all the so far reported smHDAC8/hydroxamic acid crystal structures. Besides the chelation of the zinc ion, the n-pentyl chain of J1036 is positioned in the foot pocket, which has not been observed before in the previously reported unsubstituted hydroxamic acids. Such occupancy of the foot pocket by n-alkylhydroxamic acids may offer the chance to develop selective HDAC inhibitors through further structural optimization. In addition, the newly identified hits are expected to have a lower toxicity compared to classical hydroxamic acids, as the toxicity-induced mechanism of the latter is usually caused by formation of the isocyanate via Lossen rearrangement which might be absent for n-alkyl hydroxamic acids due to substitution on the nitrogen atom [69].

In another approach, Guidi et al. performed a high-throughput assay based on the measurement of ATP in the larval stage of *S. mansoni* and screened a small library of class I HDACs inhibitors at a single concentration of 10 µM [70]. Four compounds were selected for further testing in dose response assays where they showed potency against the schistosomula in the range of 10–20 µM. Interestingly, the compounds showed variable activities against class I hHDACs in both in vitro and cell-based assays, which was not surprising taking into consideration the weak zinc binding moieties in the compounds and suggesting a potential selectivity for smHDACs. Upon testing in survival assays on adult worms, three out of the four hits (**18**–**20**) induced a significant decrease in viability of adult male worms within seven days of compound incubation at concentrations of 10 and 20 µM [70]. Since the three compounds showed a lethal action at 10 µM concentration, a sub-lethal concentration of 5 µM of compounds **19** and **20** was shown to induce a strong reduction in the number of eggs laid by worm pairs after three days of treatment. Compounds **19** and **20** were also found to induce morphological alterations in the female reproductive organs which is consistent with their effect on egg production. Finally, worm lysates treated with compound **18** and **19** showed high level of histone hyperacetylation comparable to the levels observed in case of worms treated with the pan-HDAC inhibitor TSA [70].

### 2.3. Molecular Pathways Affected by smHDAC8 Inhibitors

Both knockdown studies and the use of selective inhibitors provided a solid proof of evidence that targeting smHDAC8 is a promising antischistosomal therapy. However, the exact molecular mechanisms underlying these anthelmintic effects are yet to be revealed. This necessitated further research to identify smHDAC8 partners and substrates affected upon its inhibition and knockdown. Therefore, the interactome of smHDAC8 was analyzed using two different, but complementary, molecular biology techniques [71]. The combination of yeast two-hybrid (Y2H) screening of an *S. mansoni* cDNA library and co-immunoprecipitation (Co-IP) experiments on adult worm protein extracts identified potential smHDAC8 partner proteins involved in cytosolic and nuclear process such as DNA repair, control of metabolism, protein dephosphorylation, cell cycle regulation, and cytoskeleton organization [71]. Recently, it was suggested that smHDAC8 interaction with SmRho1.1, a schistosomal orthologue of human Ras homolog family member A (RhoA) GTPase, could represent a potential molecular mechanism by which smHDAC8 regulates the cytoskeleton. Indeed, smHDAC8 selective inhibition or knockdown were demonstrated to cause disruption of the parasite actin cytoskeleton organization, more evidently in schistosomula [72].

## 3. Schistosomal Sirtuins Are Potential Targets for Antischistosomal Therapy

As previously mentioned, the *S. mansoni* genome encodes five sirtuins expressed throughout the life cycle, with some variations in the pattern of expression [49]. To determine whether sirtuins are essential for the parasite survival, different inhibitors of human sirtuins were tested against the parasite larvae and adult worms. Indeed, all tested inhibitors induced a time and dose-dependent mortality of schistosomula. However, three Sirt1/2 inhibitors, namely, salermide, sirtinol, and MS3 caused the most significant decrease in schistosomula viability at 10 µM and killed all the larvae at 20 µM. Moreover, salermide and sirtinol were also shown to cause separation of adult worms in vitro and decrease egg production. Furthermore, salermide caused dose-dependent phenotypic changes in the gonads of adult worms demonstrated by reduction in numbers of germinal cells in the testes of males and disorganization in the ovaries of females. Interestingly, transcriptional knockdown of smSirt1 produced similar phenotypic effects in the ovaries, but not in the testis, suggesting that the phenotype observed after Salermide treatment may have resulted from inhibition of other sirtuin isoforms [49].

**Figure 4:**
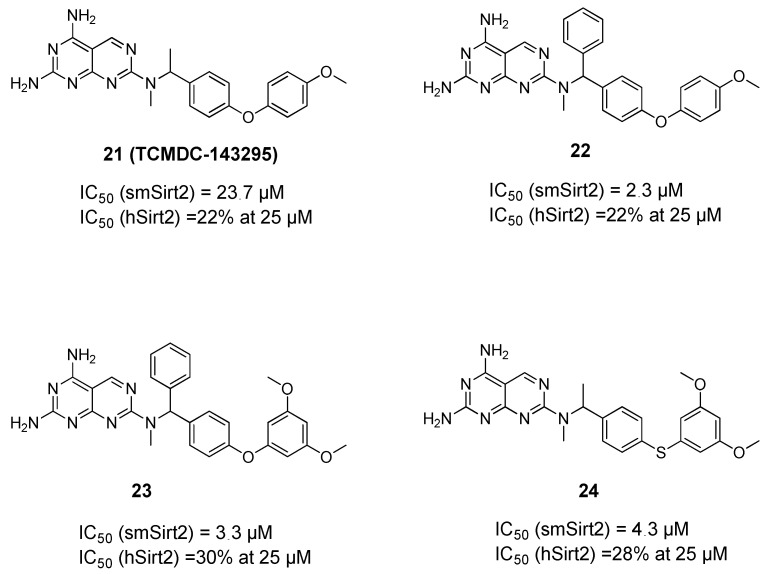
Reported inhibitors of Schistosoma mansoni sirtuins.

The above data provided a solid proof of evidence of the significance of Sirt1/2 inhibition against different life stages of the parasites. This was followed by another effort to identify novel and selective inhibitors of smSirt2 [73] based on the previous development of an in vitro assay for the determination of smSirt2 deacetylase activity [74]. Here, the GSK Kinetobox library was screened against smSirt2, and some potential hits were identified showing micromolar activity against the parasitic enzyme. One of these hits (TCMDC143295, **21**, Figure 4) was then subjected to an extensive structure–activity relationship study that resulted in compounds **22**–**24** with better enzymatic activity while also showing selectivity over human Sirt2 (hSirt2) and no general toxicity to human cells. The compounds were also demonstrated to decrease the viability of the parasite larva at 10 and 20 µM and reduce adult worm pairing and egg production [73].

## 4. Summary

Significant progress in semi-automation of phenotypic screening and drug discovery methodologies for schistosomiasis has been achieved over the last decade. Based on published data, smHDAC8 inhibitors appear to have the most promise as schistosomicidal drugs. Unfortunately, numerous compounds with good enzymatic inhibition data show no effect on the parasite, which may be due to poor bioavailability. In addition, so far little is still known about bioavailability and stability of HDACi in animal models of schistosomiasis. Most of the studies investigating the possibility of the use of HDACi for the treatment of schistosomiasis worked entirely with cultured schistosomes. Testing of HDACi in animal models of infection should bring new insights into the killing of schistosomes via HDACi and will in fact be required before considering HDACi for further preclinical trials. On the other hand, it must be mentioned that several compounds reviewed in this work should be regarded as “hits” that still need extensive optimization to be described as “leads” that could then be considered for further preclinical development. Furthermore, numerous HDAC inhibitors are currently being tested in preclinical and clinical trials for the treatment of cancer in combination with other anticancer agents. Likewise, combination therapies of smHDAC8 inhibitors and other anthelmintic agents could provide promising results. Additionally, the potential of HDACs as therapeutic targets in parasitic diseases is being actively confirmed, such as the very recent identification of *Trypanosoma cruzi* histone deacetylases 2 (tcDAC2) [75] and the development of species selective inhibitors. Therefore, the authors are confident that further HDAC inhibitors will be reported as novel antiparasitic agents in the future.

## Figures and Tables

**Table 1:** Observed interactions of the herein mentioned inhibitors in the binding site of smHDAC8 (vdW = van-der-Waals interaction).

Cpd	PDB ID	Zn^2+^-chelation *	H-bond triad **	F216 (π-π)	F151 (π-π)	K20 (cation-π)	K20 (H-bond)	H292	Y341 (π-π)	P291 (vdW)
1 (J1038)	4BZ8	X	X		X			X (H-bond)		
2 (J1075)	4BZ9	X		X		X				
3	6GXW	X	X			X		X (vdW)	X	
4	6GXU	X	X	X				X (**π-π**)		
5 (TH31)	5FUE	X	X			X	X	X (H-bond)	X	
6 (TH65)	6HTH	X	X			X	X	X (H-bond)	X	X
8	7P3S	X	X			X		X (**π-π**)	X	
12	6TLD	X	X					X (H-bond)		
13	6HU3	X	X					X (vdW)	X	X

* Bidetate chelation of catalytic zinc ion. ** Three hydrogen bonds with H141, H142, Y341.

## Data Availability

Not applicable.
